# Cross-Linked Networks of 1,6-Hexanedithiol with Gold Nanoparticles to Improve Permeation Flux of Polyethersulfone Membrane

**DOI:** 10.3390/membranes12121207

**Published:** 2022-11-29

**Authors:** Xiaoqin Niu, Yuhong Chen, Haobin Hu

**Affiliations:** 1 College of Chemistry and Chemical Engineering, Longdong University, Qingyang 745000, China; 2 School of Science, Lanzhou University of Technology, Lanzhou 730050, China

**Keywords:** membrane, surface modification, gold nanoparticles, permeability

## Abstract

It is a great challenge to design and prepare polymeric membranes with excellent permeability and good rejection. In this study, a modifier of gold nanoparticles for crosslinking and self-assembly by 1,6-hexanedithiol is fabricated and used to modify the polyethersulfone membrane as an additive, which forms a uniform porous membrane by liquid–liquid phase conversion technology. The morphology of the membrane is investigated by scanning electron microscopy (SEM), the change of the hydrophilicity of the membrane surface after modification is measured by the contact angle goniometer, and the performance of the fabricated membrane is measured by evaluating the pure water flux and the rejection ratio of bovine serum albumin. The results indicate that the permeability of the modified membrane has a significant improvement. When the mass fraction of the modifying agent is 5 wt%, the water flux of the modified membrane reaches up to 131.6 L m^−2^ h^−1^, and has a good rejection ratio to bovine serum albumin. In short, this work plays an important role in improving the flux of the membrane and maintaining good separation performance.

## 1. Introduction

In recent years, in view of the increasingly serious problems of energy shortage and environmental pollution, membrane separation technology has become an important technology to solve these problems, and is mainly used in the industries of seawater desalination, wastewater treatment, and petrochemical, etc. [1,2,3,4,5,6]. Generally, the types of familiar membrane materials include inorganic membranes and organic membranes. Polymeric membranes are widely utilized because of their low cost, ease of processing, good mechanical properties, high chemical stability, and oxidative stability [7,8]. Polymer membrane materials include polyvinylidene fluoride, polyimide, polyacrylonitrile, polysulfone, polyethersulfone, etc. Among these polymer materials, polyethersulfone has been receiving growing attention due to its excellent thermal and oxidative stability, ease of forming membrane, and other properties [7,9,10,11]. However, the inherent hydrophobicity of PES makes it easy to adsorb organic molecules such as proteins, which limits the performance and practical application of PES membranes [12,13,14]. Therefore, it is necessary to investigate modifications of PES membranes to develop high permeability of water and good rejection.

Some modification methods have been developed for improving the permeability of polymer membrane in recent years, such as chemical surface modification and blending methods [15,16,17,18]. The chemical surface modification refers to the introduction of some strongly polar groups or grafting of hydrophilic molecular groups on the surface of the membrane through chemical treatment to change the membrane surface from non-polar to polar, thereby improving the hydrophilic performance of the membrane. Although this method can improve the permeability of the membrane, the steps of modification of the membrane increase in the preparation process, which are difficult to achieve in large-scale industrial production. Besides, the bulk structure, the separation performance, and the mechanical strength of the membrane are all damaged in the complex technological process [19,20,21,22]. Compared with chemical surface modification, blending modification offers many advantages including more efficient, economical, and limited processes, and is massively used in the industry.

Functional inorganic nanoparticles, including nano-alumina, nano-silica, nano-titanium oxide, graphene nano-materials, etc., and hydrophilic polymer, including polyvinyl pyrrolidone, polyvinyl alcohol, polyethene glycol, etc., have been reported as additives to blend with membrane material in the preparation [23,24,25,26,27]. Although introducing the hydrophilic additive can make the permeation property of the membrane have a remarkable improvement, the molecular structure of the polymer is hard to control. Besides, the structure of the pores changes, and the characteristics of the membrane separation processes are affected by the hydrophilic polymer. The permeation and stability of the modified membrane is not ideal because the binding ability between matrix materials and inorganic nanoparticles is weak [28,29,30,31].

There are two important parameters, permeate flux and rejection efficiency, for identifying the separation performance membrane. Permeate flux primarily relies on the hydrophilia of the skin layer of the modified membrane, and pore size, pore number distribution, and pore area distribution. By improving the hydrophilicity of the top surface and increasing the pore size and number distribution of the membrane, the permeable flux of the modified membrane can be significantly increased. Meanwhile, the pore size of the membrane separation layer plays a decisive role in the rejection ratio. From the above, the improved performance of the membrane is mainly attributed to adjust the structure morphology and pore size of the modified membrane separation layer [32,33,34,35,36].

Herein, the modifier of gold nanoparticles of crosslinking and self-assembly by 1,6-hexanedithiol was prepared and used as a suitable additive to effect the PES membrane, to obtain a narrow pore distribution membrane by liquid–liquid phase conversion process. Because of the addition of the modifier, the hydrophilicity of the casting solution increased, and introduction of a suitable additive to the casting solution was a convenient and efficient method. In this work, numerous sponge-like pores in the matrix of the membrane were formed; this is reported to improve the separation efficiency, selectivity, and service life of the separation membrane. We controlled the number of pores in the modified membrane by changing the concentration of the 1,6-hexanedithiol.

## 2. Experimental

### 2.1. Chemicals

Trisodium citrate dihydrate and chloroauric acid were purchased from Tianjin Guangfu Fine Chemical Research Institute (Tianjin, China). Sodium borohydride was purchased from Shanghai Zhongtai Chemical Reagent Co., Ltd. (Shanghai, China). 1,6-hexanedithiol was purchased from Shanghai Macklin Biochemical Co., Ltd. (Shanghai, China). Polyethersulfone (PES, Ultrason E6020P, *Mw* = 58,000 Da) was purchased from BASF (Ludwigshafen, Germany). *N*,*N*-dimethylacetamide (DMAc; AR, purity 99.0%) was purchased from Sinopharm Chemical Reagent Co., Ltd. (Shanghai, China) and used as the solvent. Bovine serum albumin was purchased from Aladdin Reagent (Shanghai, China). Ultrapure water was made in laboratory.

### 2.2. Preparation of Gold Nanoparticles and Au-HET

Gold nanoparticles were prepared with a reduction of chloroauric acid by trisodium citrate dihydrate and sodium borohydride. In a reaction, 6 mL chloroauric acid solution was diluted by 160 mL ultrapure water. Then 6 mL (1%) trisodium citrate dihydrate solution and 6 mL (0.11%) sodium borohydride solution were sequentially added to the mixed solution under stirring at 4 °C for 30 min. After the solution returned to room temperature, 0.08 g of 1,6-hexanedithiol was quickly added to 160 mL of gold nanoparticle solution for 1 h, and dried in a 40 °C blast drying oven for 24 h to obtain the modifier Au-HET.

### 2.3. Preparation of PES and PES/Au-HET Membrane

The Au-HET of a certain mass fraction was added into DMAc. After Au-HET were completely dispersed, a certain mass fraction of PES was added into the mixture, which was stirred for 12 h at room temperature to obtain a homogeneous solution. After that, the uniform casting solution was degassed for 1 h and was spread on a clean glass surface. This was followed by spin-casting for homogeneous thickness, and the modified membrane of Au-HET was obtained by the method of liquid–liquid phase separation at room temperature. Then the modified membrane was dipped into ultrapure water for 48 h at room temperature to remove residual solvent and dried under vacuum for 48 h. In this work, the modified membranes containing Au-HET of 0, 2, 3, and 5 wt% were named M-PES, M-PES/Au-HET-2, M-PES/Au-HET-3, and M-PES/Au-HET-5.

### 2.4. Characterization

The morphologies of Au-HET were observed by transmission electron microscopy (TEM, TECNAI TF20), FTIR analysis was conducted using the Thermo-Nicolet 6700 P FTIR Spectrometer (Thermo Fisher Scientific, Madison, WI, USA), and the chemical characterizations of Au-HET were characterized by X-ray photoelectron spectroscopy (XPS, XSAM800, KRATOS Co., Ltd., Manchester, UK). The morphologies of the modified membranes were observed by scanning electron microscope (SEM, JSM-6700F JEOL, JEOL Ltd., Tokyo, Japan). The membranes were frozen in liquid nitrogen and were then broken and sputtered with a gold layer before SEM analysis. The static contact angles of the modified membrane were tested by the contact angle goniometer (WCA, PHS-3C, Precision Science Co., Ltd., Shanghai, China).

### 2.5. Membrane Performance Test

The performance of filtration and anti-fouling of the membrane were tested by a cross-flow filtration machine having an effective area of 23.7 cm^2^ at room temperature. Firstly, obtaining a steady filtration result, the modified membranes were immersed in ultrapure water for 1 h and were pre-compacted by ultrapure water for 30 min at 0.25 MPa. The first-time pure water flux (*J*_*w*1_, L m^−2^ h^−1^) of the membrane was measured for 1 h at 0.2 MPa and was computed by Equation (1). After that, the Bovine serum albumin (BSA) solution flux *(J_p_*) was measured in the BSA aqueous solution (1.0 mg mL^−1^) for 1 h at 0.2 MPa. Finally, the membranes were rinsed by ultrapure water for 1 h. The second-time pure water flux (*J*_*w*2_) was measured again for 1 h at 0.2 MPa. The flux recovery ratio (FRR) and total fouling ratio (*R_t_)* were computed by Equation (2) and Equation (3), respectively. The reversible flux decline ratio (*R_r_)*, and the irreversible flux ratio *(R_ir_)* were respectively computed by Equations (4) and (5).

(1) $$J_{w}=\frac{V}{St}\;$$

(2) $$FRR=\frac{J_{w2}}{J_{w1}}\times 100\;\%$$

(3) $$R_{t}=\left(1-\frac{J_{p}}{J_{w1}}\right)\times 100\;\%$$

(4) $$R_{r}=\frac{J_{w2}-J_{p}}{J_{w1}}\times 100\;\%$$

(5) $$R_{ir}=\left(1-\frac{J_{w2}}{J_{w1}}\right)\times 100\;\%\;$$

In these formulas, V (L), S (m^2^), t (h), *J*_*w*1_, *J*_*w*2_, and *J_p_* respectively correspond to the volume of permeated water, the effective membrane area, the operation time, the first-time water flux (L m^−2^ h^−1^), the second-time water flux (L m^−2^ h^−1^), and the flux of the BSA solution (L m^−2^ h^−1^).

Molecules of different size were separated by the modified membrane and the rejection coefficient (R) was computed by Equation (6).

(6) $$R=\left(1-\frac{C_{p}}{C_{f}}\right)\times 100\%$$

In the formula, *C_p_* and *C_f_* (mg mL^−1^) respectively correspond to the concentration of the permeated and the BSA solution, which were measured by ultraviolet-visible (UV-vis) spectroscopy.

## 3. Results and Discussion

**Figure 1:**
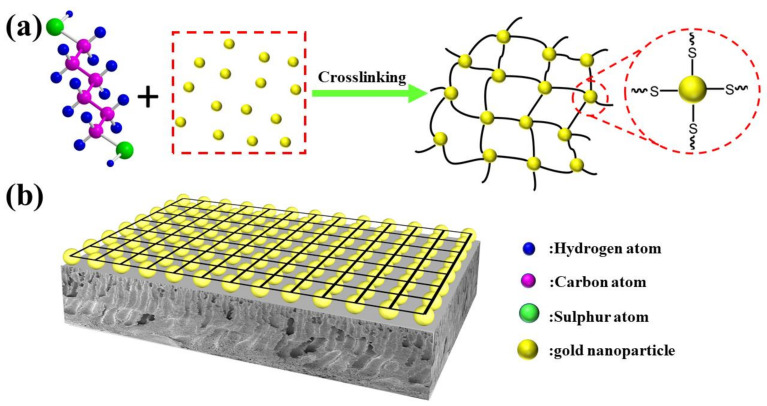
Schematic illustration of fabricating (**a**) the crosslinked gold nanoparticles (**b**) modified membrane.

The preparation of the modified PES membrane is schematically demonstrated as Figure 1, involving the following steps: preparing the gold nanoparticles with a reduction of chloroauric acid by trisodium citrate dihydrate and sodium borohydride, preparing the cross-linked networks of 1,6-hexanedithiol and gold nanoparticles (CLN), and blending CLN with PES for preparing modified membrane (CLN-PES). 1,6-hexanedithiol and gold nanoparticles react due to the functional thiol groups in 1,6-hexanedithiol and the citric based part of gold nanoparticles. The bi-functional thiol groups make the cross-linked case of the two things and form CLN. When the modifier of CLN is blended with PES, it is assembled in the surface of membrane.

**Figure 2:**
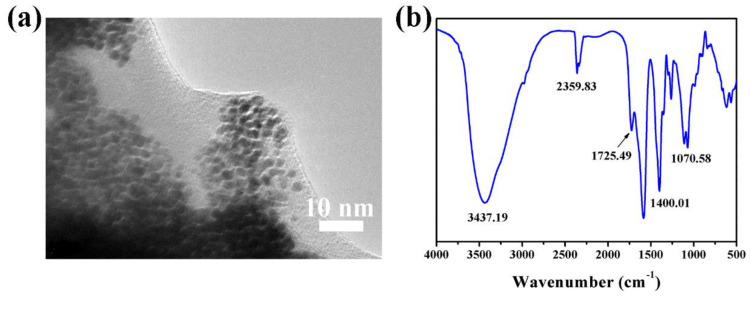
(**a**) TEM image of Au-HET, and (**b**) FTIR spectrum of Au-HET.

The morphology and structure of Au-HET are characterized by TEM and the FTIR as shown in Figure 2. From the TEM image in Figure 2a, one can find that the diameter of gold nanoparticles in Au-HET is about 5 nm, which is no different from gold nanoparticles as prepared. At the same time, after 1,6-hexanedithiol is added into the solution of gold nanoparticles and the reaction system is dried, the gold nanoparticles have not agglomerated. According to the Figure 2b, several characteristic vibrational bands of Au-HET can be seen: hydroxide radical -OH stretch at 3437 cm^−1^, C=O stretch at 1725 cm^−1^, CH_2_ is bent at 1400 cm^−1^, and CH_2_-O stretch at 1070 cm^−1^. According to the information reported, the S-H terminal group that should has a stretch peak at about 2654 cm^−1^ does not appear, indicating the S-H terminal group has cooperated on the surface of nanoparticles [37,38].

**Figure 3:**
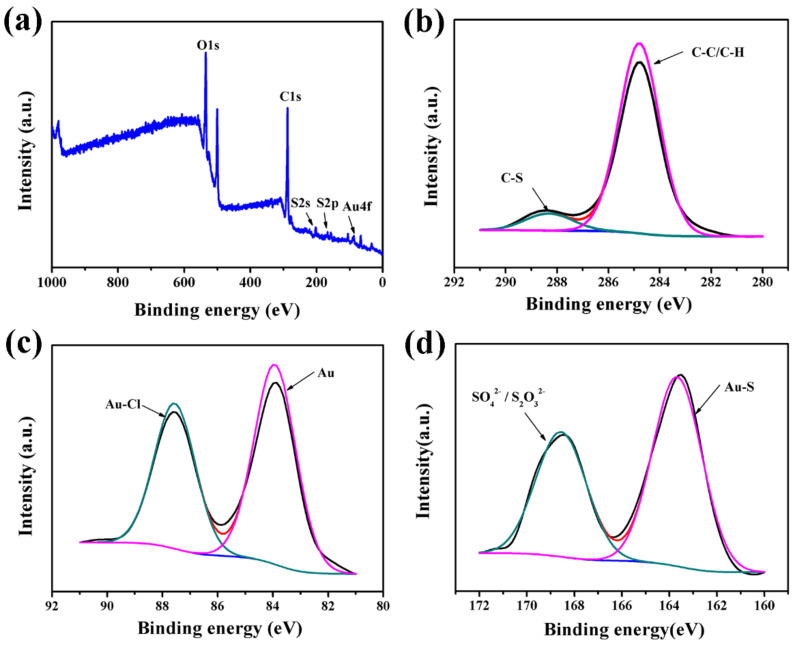
XPS spectra of Au-HET: (**a**) the peaks of Au 4f, S 2p, S 2s, C 1s, and O 1s; (**b**) the peaks of C 1s, C-C/C-H and C-S; (**c**) the peaks of Au; (**d**) the peaks of Au-S.

In Figure 3a, the peaks for Au 4f, S 2p, S 2s, C 1s, and O 1s can be identified at ca. 84, 168, 206, 288, and 532 eV, respectively, in the measurement of X-ray photoelectron spectroscopy (XPS) of Au-HET. To further obtain the details of structures and compositions of Au-HET, the results were studied utilizing XPS-peak-differentiating analysis. The peaks of C 1s are shown in Figure 3b, and the C on XPS is fitted into two peaks centered at 284.8 and 288.3 eV, which are attributed to the C-C/C-H and C-S species, respectively. In addition, we can find the peaks of Au and Au-S from Figure 3c,d. All the results of XPS of Au-HET further indicate that the thiol has connected on the surface of the gold nanoparticles.

**Figure 4:**
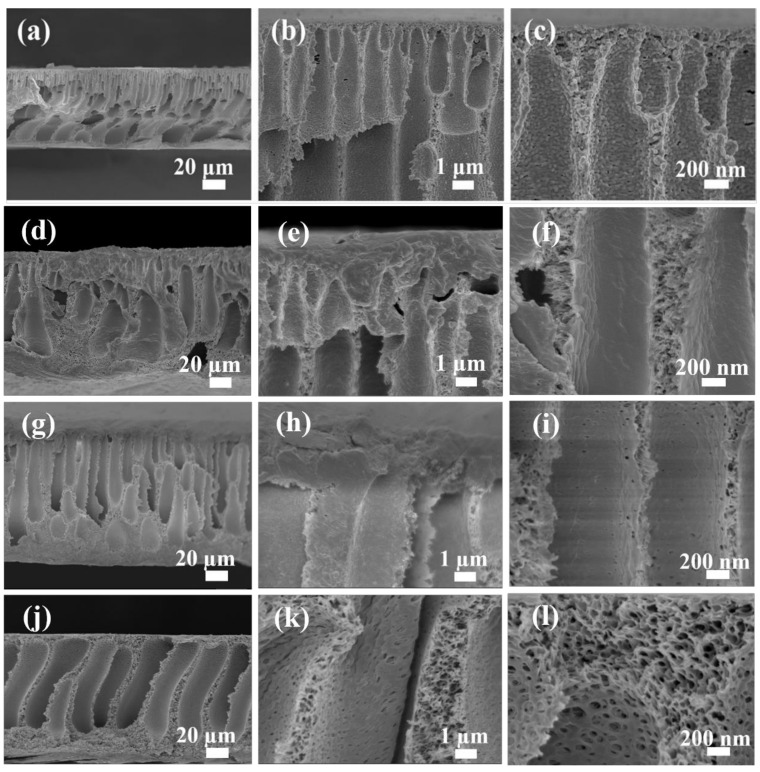
SEM images of cross-sectional views of (**a**–**c**) M-PES, (**d**–**f**) M-PES/Au-HET-2, (**g**–**i**) M-PES/Au-HET-3, and (**j**–**l**) M-PES/Au-HET-5.

Figure 4 shows the cross-profile morphologies of the pristine membrane and the modified membrane. It can be seen from the figures that both the pristine membrane and the modified membranes had an asymmetrical structure. The membrane cross-section consisted of a dense layer that played the role of molecular sieve and the porous sublayer of the membrane has a finger-like pore structure. Compared with the pristine PES membrane, the dense top layer on the surface of the modified membrane became thinner, which would reduce the transmission resistance of water on the separation layer for increasing the water perviousness. In addition, the internal face of the modified membrane is constituted by a large number of sponge holes, which constituted channels among the thick finger pores and improved the permeability of the modified membrane. Also, the number and the size of sponge holes of the modified membrane increased as the content of modifying agents in the casting solution increased. This revealed that the thickness of the dense top layer, and the number, size, and structure of the finger-like pores have been controlled by the content of modifying agent in the casting solution.

**Figure 5:**
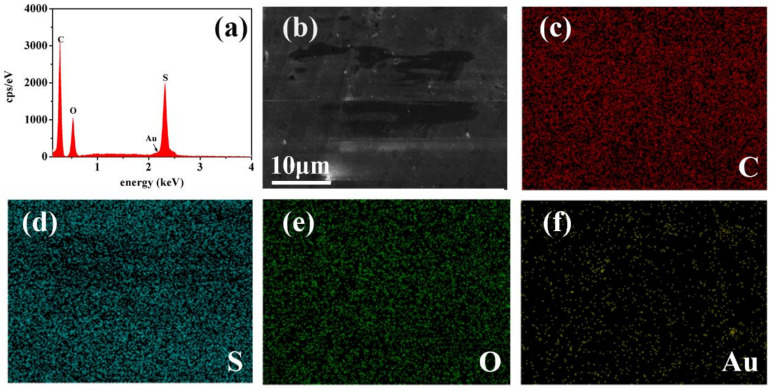
(**a**) EDS mapping of membranes of Au-HET with contents 5 wt%, and (**b**–**f**) four elements.

The results of energy-dispersive spectroscopy show that the M-PES/Au-HET consist of four elements, C, S, O, and Au (Figure 5a), with mass percentage of 66.8, 6.85, 22.59, and 0.47%, respectively, as shown at Table 1. EDS mappings of the modified membrane, M-PES/Au-HET-5, is shown in Figure 5b–f. The results further indicate that the modified membrane contains elements of C, S, O, and Au. These results show that the modifier of CLN has been introduced into the surface of the PES membrane.

**Figure 6:**
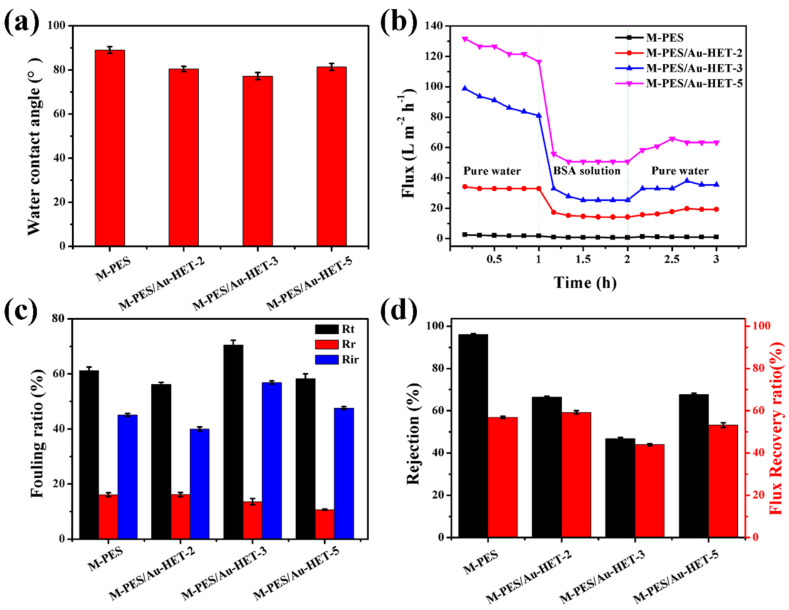
Properties of the pristine and modified membranes of Au-HET with contents of 0, 2, 3, and 5 wt%: (**a**) water contact angle, (**b**) time–dependent fluxes, (**c**) fouling ratios, and (**d**) water flux recovery ratios (FRR) and BSA rejection ratios.

To estimate the surface hydrophilicity of the membranes, the water contact angles were measured, and the results are shown in Figure 6a. Because of the hydrophobic property of PES, the static water contact angle of the pristine PES membrane was 89°. Compared with the pristine PES membrane, the water contact angle of the modified membranes reduced a little because of the formed micropores on the surface. The transfer performance of the pristine membrane and the modified membrane are shown in Figure 6b. The results show that compared with the pristine membrane, the flux of modified membranes was significantly improved, and with the content of the modifying agent increased, the flux increased gradually. It can be seen than the flux decreased when the ultrapure water was changed to BSA solution. The main reason is because the water contact angles of the modifying membrane were little changed, which showed in the surface of the modified membrane being almost as rough as the pristine membrane, and the rough membrane surface is easily adhered to by BSA protein, resulting in the decrease of water flux. The anti-fouling ability of the membranes are shown in Figure 6c. The results show that R_t_ and R_ir_ changed with the modifying agent being added. In addition, flux recovery ratio (FRR) and BSA rejection are presented in Figure 6d. The results show that the flux recovery rate of the modified membrane also changed with the modifying agent being added. According to the information in Figure 6c,d, there is no obvious regular change; the reason may be that the dosage of the additive changes too much, resulting in no regular change in the results. However, it is undeniable that the performance of the modified membrane has been significantly improved.

## 4. Conclusions

The cross-linked networks of 1,6–hexanedithiol and gold nanoparticles are fabricated via the method of reduction and cross–linking, and are used to improve the performance of PES membrane as an additive to form a uniform porous membrane by liquid-liquid phase conversion technology. The modified additives change the surface morphology and pore structure of the modified membrane, which causes the superstructure dense layer to be thinner and form a large number of micropores. In addition, the number of micropores in the modified membrane can be controlled by changing the concentration of the modifier. Moreover, the microporous structure of the separation layer would not be influenced by the modifying agent, which makes the modified membrane not only reach a high permeability but also keep a good rejection. With the concentration of Au-HET being 5 wt%, the pure water flux of the modified membrane reaches up to 131.6 L m^−2^ h^−1^, and the BSA rejection ratio maintains 67.6%, indicating that the modifying agent based on gold nanoparticles commendably improved the performance of the PES membrane.

## Figures and Tables

**Table 1:** Mass percentage of M-PES/Au-HET-5 about different elements.

Element	C	S	O	Au
*Wt* %	66.68	6.85	25.99	0.47

## Data Availability

Not applicable.
